# Hair cell toxicology: With the help of a little fish

**DOI:** 10.3389/fcell.2022.1085225

**Published:** 2022-12-13

**Authors:** Alejandro Barrallo-Gimeno, Jordi Llorens

**Affiliations:** ^1^ Department de Ciències Fisiològiques, Facultat de Medicina i Ciències de la Salut, Campus de Bellvitge, Universitat de Barcelona, L’Hospitalet de Llobregat, Spain; ^2^ Institut de Neurociències, Universitat de Barcelona, Barcelona, Spain; ^3^ Institut D'Investigació Biomèdica de Bellvitge, IDIBELL, L’Hospitalet de Llobregat, Spain

**Keywords:** hair cell, zebrafish, ototoxicity, otoprotection, cisplatin, aminoglycoside antibiotic

## Abstract

Hearing or balance loss are disabling conditions that have a serious impact in those suffering them, especially when they appear in children. Their ultimate cause is frequently the loss of function of mechanosensory hair cells in the inner ear. Hair cells can be damaged by environmental insults, like noise or chemical agents, known as ototoxins. Two of the most common ototoxins are life-saving medications: cisplatin against solid tumors, and aminoglycoside antibiotics to treat infections. However, due to their localization inside the temporal bone, hair cells are difficult to study in mammals. As an alternative animal model, zebrafish larvae have hair cells similar to those in mammals, some of which are located in a fish specific organ on the surface of the skin, the lateral line. This makes them easy to observe *in vivo* and readily accessible for ototoxins or otoprotective substances. These features have made possible advances in the study of the mechanisms mediating ototoxicity or identifying new potential ototoxins. Most importantly, the small size of the zebrafish larvae has allowed screening thousands of molecules searching for otoprotective agents in a scale that would be highly impractical in rodent models. The positive hits found can then start the long road to reach clinical settings to prevent hearing or balance loss.

## Introduction

Hair cells are specialized mechanosensory epithelial cells responsible for the transduction of sound and accelerations, important for body orientation, vision stabilization and motor control. In mammals, they are found in the cochlea and the vestibular system. The transducing part of the hair cells, as indicated by its name, is a bundle of stereocilia in their apical side that bend in response to the vibration of sound waves or the movement of fluid inside the inner ear cavities. Cilia deflection opens ion channels that lead to the depolarization of the hair cell and activate synaptic transmission to afferent neurons from the spiral (auditory) or vestibular ganglia (Ó Maoiléidigh and Ricci, 2019). Hair cells contain in their basolateral membrane a specific type of presynaptic machinery, the ribbon synapse (also present in some retinal cells), adapted to constant activity (Moser et al., 2020). Hearing or vestibular impairment are disabling conditions that represent a major impact in the lives of those afflicted, particularly children (WHO).

Hair cells are exquisitely sensitive to insults and deficits in hair cell function cause hearing loss and imbalance. This loss is irreversible, as hair cells in adult mammals have a very limited, if any, regenerative capacity (Rubel et al., 2013; Lee and Waldhaus, 2022). These deficits can be triggered by age, viral infection, or environmental insults, such as noise or chemical substances (collectively known as ototoxins), by either accidental exposure or pharmacological treatment. Among the latter, two drugs can be highlighted due to their widespread use: the chemotherapeutic agent cisplatin and the aminoglycoside family of antibiotics (Schacht et al., 2012). Effects of ototoxins can lead to the death of the hair cells or to damage in their synaptic coupling to afferent neurons (Liberman and Kujawa, 2017). Therefore, potential ototoxicity of novel drugs should be assessed before or during clinical trials to prevent harmful side effects (Coffin et al., 2021), as well as the ototoxicity of environmental pollutants (Fábelová et al., 2019). Equally important, knowledge of the mechanisms that produce ototoxicity will help in obtaining drugs that would prevent or mitigate these known ototoxic effects when inevitable.

Many proteins essential for hair cell function (cilia components, ion channels, neurotransmitter release machinery, synaptic junction proteins, transcription factors) have been found through identifying the genes responsible for congenital deafness or generating deaf animal models (Taiber et al., 2022). However, research into the normal biology of hair cells or the pathophysiological mechanisms that lead to their dysfunction has been hampered in mammals by their localization inside the temporal bone, making them difficult to reach or sample, and its complex three-dimensional architecture. Additionally, clinically relevant mammalian models of ototoxicity have been difficult to develop due to overall toxicity after systemic administration of the ototoxin (Wu et al., 2001; Murillo-Cuesta et al., 2010; Fernandez et al., 2019). There are cell lines derived from the cochlea (Weir et al., 2000; Kalinec et al., 2003), but none of them is truly generating hair cells *in vitro*, which require specific physiological conditions (i.e., a potassium-rich extracellular environment) and supporting cells to be functional. Other experimental systems to study hair cells in mammals imply the use of neonatal or adult inner ear cultures (Cunningham, 2006; Ou et al., 2013; Ogier et al., 2019), which are laborious and offer limited throughput in toxicology studies (Lim et al., 2018). These issues could be solved by forthcoming advances in organoid research (Nist-Lund et al., 2022).

Mechanosensory cells are present throughout the animal kingdom, and hair cells homologous to those found in mammals have been observed in all vertebrate groups (Coffin et al., 2004). Ototoxicity has been studied in birds (Park and Cohen, 1982) and, unlike mammals, their hair cells show regenerative capacity (Cruz et al., 1987). The inner ears of birds are similar to those of mammals, and present the same problem: difficult access and limited throughput. Hair cells in zebrafish larvae are also able to regenerate after injury (Williams and Holder, 2000; Harris et al., 2003), and they offer two key advantages for ototoxicology research: accessible hair cells and a small size that allows upscaling.

**FIGURE 1:**
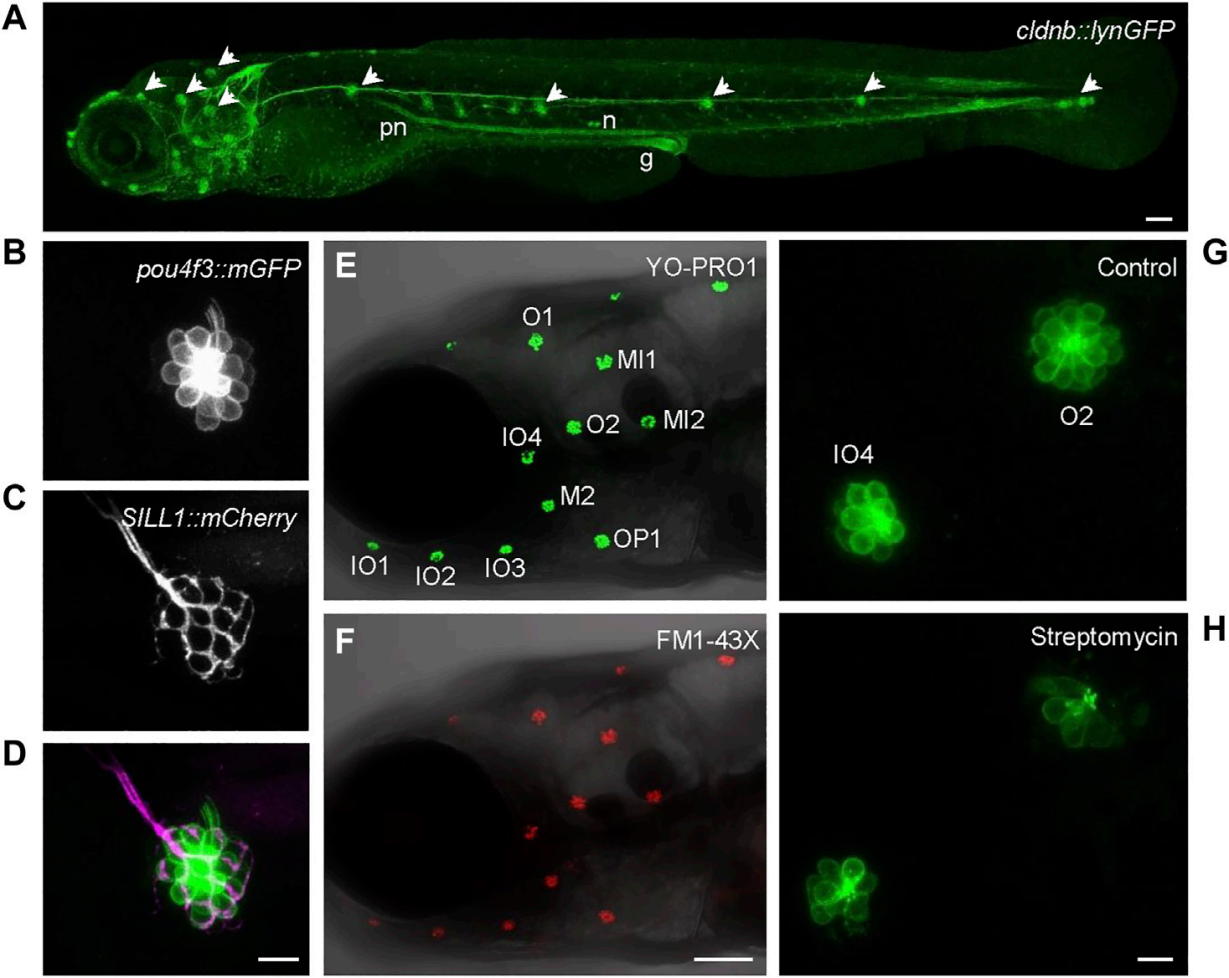
Images of hair cells in 5dpf zebrafish larvae. **(A)** Composite image obtained with laser scanning microscopy of a transgenic larva expressing *cldnb::lynGFP* (Haas and Gilmour, 2006), gift of Paola Bovolenta (CSIC, Madrid). Arrowheads point to some of the neuromast of the anterior (head) and posterior (trunk) lateral line. pn, pronephric duct; n, nephrons; g, distal gut. **(B–D)** Detail of an individual neuromast of the posterior lateral line showing **(B)** hair cells expressing the transgene *pou4f3::mGFP* (Obholzer et al., 2008), gift of Berta Alisa (Univ. Pompeu Fabra, Barcelona); **(C)** its afferent innervation expressing the transgene *SILL1::mCherry* (Pujol-Martí et al., 2012), gift of Hernán López-Schier (Helmholtz Zentrum, Munich); **(D)** overlay of the previous images. **(E,F)** Overview of the anterior lateral line showing hair cells labelled with **(E)** YO-PRO1 and **(F)** FM1-43X; neuromasts are labelled according to (Raible and Kruse, 2000). Inner ear hair cells are not labelled with these dyes; otoliths are visible behind neuromasts O2 and MI2. **(G,H)** Image of two anterior lateral line neuromasts labelled with the transgene *pou4f3::mGFP* under standard conditions **(G)** or after treatment with the aminoglycoside antibiotic streptomycin **(H)**. Scale bar in A, E, **(F)** 100 μm; scale bar in **(B,C,D,G,H)** 10 μm. All images taken in the Microscopy Unit of Centres Científics i Tecnològics de la Universitat de Barcelona (CCiTUB) at the Campus de Bellvitge.

Zebrafish hair cells are found in the inner ear and in a fish-specific organ, the lateral line (Figure 1A). This organ is responsible for sensing water flow in the surface of the fish (Suli et al., 2012), for detecting prey or predators (McHenry et al., 2009) and for schooling behavior (Venuto et al., 2022). The lateral line extends around the head (anterior lateral line, aLL) and along the rest of the body (posterior lateral line, pLL) and is formed by a series of structures called neuromasts, which consist of groups of hair cells and supporting cells (Ghysen and Dambly-Chaudière, 2007; Thomas and Raible, 2020). The lateral line is functional at 4–5 days post-fertilization and the biophysical properties of hair cells in zebrafish larvae at that stage have been characterized (Olt et al., 2014).

While the hair cells in the inner ear of the zebrafish larvae are located inside the otic capsule (Alsina and Whitfield, 2017), lateral line hair cells are exposed to the aquatic medium and are thus accessible for substances dissolved therein, like chemical agents whose toxic or protective effect on hair cells needs to be assessed (Ton and Parng, 2005). The superficial localization of the lateral line in the zebrafish larvae allows for the observation of hair cells *in vivo*. They can be labelled with fluorescent dyes added to the medium for a short time, like Yo-Pro1 (Figure 1E), FM1-43 (Figure 1F), DASPEI or DAPI (Baxendale and Whitfield, 2016). Alternatively, many transgenic zebrafish lines express fluorescent proteins or genetically-encoded indicators under the control of hair cell-specific promoters, such as *pou4f3* (Figure 1B; Xiao et al., 2005), *myo6b* (Obholzer et al., 2008) or *pavlb3a/b* (McDermott et al., 2010), that allow their observation *in vivo* for extended periods (Zhang and Kindt, 2022). Lateral line hair cell function can be measured directly by stimulation with a micro water jet (De Faveri et al., 2021). Indirect measurements are possible as well, as perturbations of the lateral line function will alter zebrafish larvae swimming behavior in a water current (Suli et al., 2012; Niihori et al., 2015; Todd et al., 2017; Tantry et al., 2022), even in an ototoxin-specific manner (Newton et al., 2022). The lateral line is also responsible for the larval startle response, an avoidance reflex upon perturbation of their surroundings (Buck et al., 2012). Some of the first genes affecting hair cell function in zebrafish were identified because the defective larvae swam in circles, hence called circler mutants (Nicolson et al., 1998).

Zebrafish offer other advantages as an experimental animal model, such as being more amenable to genetic manipulation, allowing loss-of-function analysis or generation of transgenic animals (Holtzman et al., 2016). An individual healthy female zebrafish can lay more than a hundred eggs on a weekly basis, and systems for multiple spawning exist that can yield thousands of eggs simultaneously (Adatto et al., 2011); therefore, obtaining embryos for toxicological testing is not an issue, even in small facilities. As the use of animals in experimental research should adhere to the 3Rs (Replacement, Reduction and Refinement) principle following Directive 2010/63/EU in the European Union, there is another advantage on the use zebrafish larvae for toxicological studies: they are not constricted by the regulation on animal experimentation. It should be noted, however, that these criteria might vary between countries and even different institutions in the same country (i.e., US). Zebrafish larvae are considered experimental animals in the European Union only from 5 days post fertilization (dpf) on, when they can feed on their own (Strähle et al., 2012; Moulder, 2016), and by that moment, the hair cells of the lateral line are already developed and functional. Therefore, zebrafish larvae represent an *in vivo* model with the advantages of an *in vitro* system for high-throughput toxicological studies focused on hair cells or other organs (Cassar et al., 2020; MacRae and Peterson, 2022).

This work will review recent advances in ototoxicology using the zebrafish as experimental model and its potential as a tool for the discovery of drugs with ototoxicity and drugs that would prevent hair cell damage.

## Cisplatin ototoxicity in zebrafish

Cisplatin (*cis*-dichlorodiammineplatinum (II)) is an antineoplasic agent widely used in adults and children (Ranasinghe et al., 2022). Upon entering the cell, it binds to DNA, creating platinum covalent adducts which damage DNA, triggering DNA-repair response, oxidative stress and subsequent apoptosis (Riddell and Lippard, 2018; Gentilin et al., 2019). Despite its effectiveness against solid tumors, cisplatin presents with high prevalence dose-limiting adverse side effects due to its action in non-proliferating cells; it is toxic to liver, kidney, nervous system and the cochlea, affecting hair cells and spiral ganglion neurons (Callejo et al., 2015; Rybak et al., 2019; Prayuenyong et al., 2021). Close to half a million cases of hearing loss associated to the treatment with cisplatin per year globally have been estimated (Dillard et al., 2022).

Cisplatin was found to cause hair cell death in the lateral line of zebrafish larvae in a dose-dependent manner (Ton and Parng, 2005; Ou et al., 2007). Cisplatin effects on adult zebrafish have been less studied, but it does produce hair cell damage in the lateral line and changes in their swimming behavior (Kim et al., 2014). In addition to hair cells, cisplatin also affects the embryonic kidney as in mammals (Hentschel et al., 2005) and other cell types in the skin, the ionocytes, responsible for ionic homeostasis (Hung et al., 2019). Ionocytes have been found recently to migrate inside the neuromasts of zebrafish larvae in certain environmental conditions, highlighting the relevance of ionic homeostasis for hair cell function (Peloggia et al., 2021). On the other hand, mutations in two genes that play a role in ionic homeostasis, the chloride/bicarbonate exchanger slc4a1b and the transcription factor gcm2, provide some resistance to cisplatin- and aminoglycoside-induced damage to hair cells (Hailey et al., 2012; Stawicki et al., 2014).

The manner in which cisplatin enters the hair cells is not completely elucidated. It had been proposed to involve passive diffusion through the plasma membrane after substitution of the two chloride atoms by hydroxyl groups, and through two transporters at the plasma membrane, copper transport 1 (CRT1) and organic cation transporter 2 (OCT2) (Riddell and Lippard, 2018; Gentilin et al., 2019). Zebrafish hair cells lack *Oct2* expression and show widespread expression of *Crt1* (McDermott et al., 2007; Thomas et al., 2013). Using a fluorescently-labeled cisplatin analog (Rho-Pt) in zebrafish larvae, and a mix of pharmacological and genetic tools to inhibit mechanotransduction in hair cells, it was demonstrated that the mechanotransduction channel (MET) is required for cisplatin uptake and death of hair cells (Thomas et al., 2013). Recently, this has been proved the case also in mice; cochlear explants from mice lacking Tmie (one of the proteins that form the mechanotransduction channel) are partially resistant to cisplatin treatment and Oct2 inhibition further increase their protection (Li et al., 2022).

Upon entering the hair cell, cisplatin causes vacuolization, condensation of chromatin and, specially, degeneration of mitochondria (Giari et al., 2012). Mitochondria have a central role in ototoxin induced hair cell death, because of their energy-demanding activity and the production of reactive oxygen species (ROS) (Holmgren and Sheets, 2020). Treatment with mdiv-1, a proposed inhibitor of mitochondrial fission, a process indicative of mitochondrial dysfunction and excess production of ROS (Smith and Gallo, 2017), reduced cisplatin damage to hair cells in zebrafish (Vargo et al., 2017). Cisplatin treatment of zebrafish larvae labeled with fluorescent indicators of mitochondrial membrane potential or a genetically-encoded calcium indicator showed that cisplatin increased mitochondrial membrane hyperpolarization, calcium levels and ROS production in hair cells, leading to the activation of caspase 3 and apoptosis (Lee et al., 2022). These authors noted a heterogeneous response to cisplatin even among hair cells of the same neuromast, that could be related to the fact that not all hair cells are active simultaneously, and some are synaptically silent and would show less energy consumption (Zhang et al., 2018). Elevated ROS levels from dysfunctional mitochondria lead to an increase in protein nitration (Nakamura and Lipton, 2020), and cisplatin also induced this process in zebrafish hair cells, with a reduction in the levels of Lmo4, a transcriptional regulator (Shahab et al., 2021).

Besides the evidences linking cisplatin to mitochondria dysfunction, other pathways have been implicated in cisplatin-induced hair cell damage. Cisplatin activates Toll-like receptor 4 (TLR4) at the plasma membrane in a similar manner to the lipopolysaccharide (LPS) found in Gram-negative bacteria (Babolmorad et al., 2021). Although fish show a different response to LPS than mammals, and some teleost species have lost the *Tlr4* gene, zebrafish has two ortholog genes, *tlr4a* and *tlr4b* (Sepulcre et al., 2009) and reducing their expression with morpholino antisense oligonucleotides attenuates cisplatin effects on hair cells (Babolmorad et al., 2021).

## Aminoglycoside ototoxicity in zebrafish

The aminoglycoside family of antibiotic comprises a series of structurally related compounds of both natural and semisynthetic origin; to name a few: gentamicin, streptomycin, kanamycin and neomycin. They are used primarily to treat infections by Gram-negative bacteria, like those linked to cystic fibrosis, urinary tract or neonatal infections. Due to their cationic nature, aminoglycosides have poor gastrointestinal absorption, and they are administered intravenously or intramuscularly (Krause et al., 2016; Kim et al., 2022). Inside bacteria, aminoglycoside antibiotics bind to ribosomes and interfere with translation, leading to defective proteins and death of the bacteria (Hermann, 2007). Aminoglycoside antibiotics have been long known for their side effects, mainly nephrotoxicity and ototoxicity (Kim et al., 2022). While it has been proposed that aminoglycosides would enter renal cells through endocytosis mediated by megalin (Nagai and Takano, 2014), entry into hair cells depends on the activity of the mechanotransduction channel (Marcotti et al., 2005; Alharazneh et al., 2011). Nevertheless, megalin has been implicated in aminoglycoside transport into the cochlea as well (Kim and Ricci, 2022). Once inside the cell, aminoglycosides accumulate in lysosomes (Hashino et al., 1997) and cause mitochondrial dysfunction and production of ROS, leading to hair cell death (Böttger and Schacht, 2013), although the precise mechanism remains elusive.

Aminoglycoside ototoxic effects on hair cells of the zebrafish larvae were identified more than 20 years ago (Seiler and Nicolson, 1999; Williams and Holder, 2000), as well as the potential of this system to study its rapid hair cell regeneration after injury (Harris et al., 2003). Zebrafish hair cell susceptibility to neomycin depends on their developmental or maturation stage, being more sensitive at 5dpf than at 4dpf (Murakami et al., 2003; Santos et al., 2006). Another factor to be considered is the calcium concentration in the medium used to incubate the zebrafish larvae: elevated calcium concentrations protect to some extent from neomycin and gentamicin-induced hair cell damage (Coffin et al., 2009). Strikingly, and relevant for the reproducibility of toxicological research, there are differences in aminoglycoside sensitivity among wild-type zebrafish strains (Wiedenhoft et al., 2017).

Members of the aminoglycoside family of antibiotics differ in their effects on zebrafish hair cells, in terms of doses and time required to induce their death: neomycin is faster and more effective than gentamicin, streptomycin (Figures 1G,H) or kanamycin; also, gentamicin-induced hair cell death persisted after washout, unlike with neomycin (Owens et al., 2009). These data suggested that there may be multiple pathways to aminoglycoside-induced hair cell death. This difference between neomycin and gentamicin was also shown using pharmacological and genetic modulators of cell death (Coffin et al., 2013a; Coffin et al., 2013b).

One of the advantages of the use of zebrafish to study hair cells is their accessibility for observation. Fluorescently-labelled aminoglycosides have been used to show their entry in hair cells *in vivo* through endocytic and non-endocytic pathways, the involvement of the mechanotransduction channel, and accumulation in lysosomes (Wang and Steyger, 2009; Hailey et al., 2017). It is worth noting that labelled gentamicin, which is less rapid in killing hair cells, accumulates faster in lysosomes than labelled neomycin, which induces hair cell death more quickly (Hailey et al., 2017), suggesting aminoglycoside handling inside the cell has a role in their ototoxicity. Another interesting difference between neomycin and gentamicin modes of action in hair cells is found in their transport from the proposed point of entry, the tips of the sterocilia, to the cytoplasm. Mutations inactivating proteins involved in retrograde intraflagellar transport provide resistance towards neomycin-induced hair cell death, but not so much against gentamicin (Stawicki et al., 2016; Stawicki et al., 2019).

The role of mitochondria in hair cell death after aminoglycoside treatment has been extensively studied in zebrafish larvae. Morphological alterations in mitochondria appear already at low concentrations and short exposures to neomycin, even at subtoxical doses; loss of mitochondrial potential was observed at higher doses (Owens et al., 2007). A mutation on the mitochondrial protein Mpv17 impairs mitochondrial calcium levels and raises their ROS production; although these fish are viable and show no behavioral phenotype, their hair cells are more sensitive to aminoglycoside-induced damage (Holmgren and Sheets, 2021). Other studies used a collection of genetically encoded calcium indicators specifically expressed in hair cells to follow the changes in the concentration of this ion *in vivo* after aminoglycoside treatment; the data showed that neomycin caused a rise in the calcium levels in the mitochondria and the cytosol (Esterberg et al., 2013; Esterberg et al., 2014) and then, an increased oxidation and the production of ROS (Esterberg et al., 2016). Hair cell function is energy demanding, and the associated mitochondrial sustained activity to supply the required energy imposes a toll on hair cell susceptibility to aminoglycosides, altering mitochondrial turnover and repair (Pickett et al., 2018). One cellular process that is responsible for energy demand is synaptic transmission between hair cells and their afferent neurons, as genetic or pharmacological blocking of synaptic transmission in hair cells provides protection from aminoglycoside (both neomycin and gentamicin)-induced cell death, precisely by reducing mitochondrial oxidation and accumulation of ROS (Lukasz et al., 2022).

## Screening for potential ototoxins

Beyond studying the mechanism behind well-known ototoxins, zebrafish larvae are useful to discover potential ototoxic effects of other molecules more rapidly than other animal studies assessing auditory end points. A systematic approach was taken to screen more than a 1000 compounds already approved by the American Food and Drug Administration (FDA) for medical use, and it found fourteen new potential ototoxins (Chiu et al., 2008). Nevertheless, this study had some limitations, as it tested only one relatively short exposure time (1 h) and some ototoxins need more time to cause detectable hair cell damage; precisely one of such is gentamicin, which failed to test positive in this screen. Automation of the inspection of stained hair cells would allow to refine such high throughput screening process to acquire more sensitivity (Philip et al., 2018).

More focused approaches targeting specific classes of drugs have been performed using the zebrafish larvae. In one of the most recent examples, the COVID-19 global pandemic has brought up the necessity of rapidly developing therapies to reduce its effects. This need for speed may lead to the use of drugs without the proper consideration of potential side effects, like ototoxicity. Some of the proposed therapies for COVID-19 (having real benefits or not) have been found to be toxic for zebrafish larvae hair cells, like hydroxichloroquine or ivermectin (Davis et al., 2020; Coffin et al., 2022). A screen of more than 500 natural products that can be found on dietary herbal supplements found nine new potential ototoxins (Neveux et al., 2017). Although cisplatin is an antitumoral drug well known for its ototoxic effects, another five drugs used to treat cancer have been shown to have similar ototoxic side-effects, as well as potential synergistic effects among them (Hirose et al., 2016). The issue of the potential synergistic effects of drugs being administered simultaneously is important, as many patients present comorbidities that need treatment. Glucocorticoids have been proposed to treat hearing loss (Meltser and Canlon, 2011). However, using zebrafish larvae it was demonstrated that previous exposure to glucocorticoids like cortisol or dexamethasone increased neomycin damage to hair cells (Hayward et al., 2019; Saettele et al., 2022). On a positive note, testing the ototoxicity of new semisynthetic aminoglycoside antibiotics has shown that etimicin exerts less damage to lateral line hair cells in zebrafish larvae than gentamicin (Shao et al., 2021), so this antibiotic could become an alternative for the ototoxic members of the family.

Another potential field of application is the detection of ototoxicity in environmental samples, contamination of which could be relevant for both human health and aquatic habitats. Heavy metals like copper, cadmium or gadolinium are known to be toxic to zebrafish hair cells (Hernández et al., 2006; Linbo et al., 2009; Rah et al., 2018; Schmid et al., 2020). Other chemical compounds that have been found to induce hair cell damage in zebrafish larvae include: pesticides (Nasri et al., 2016; Wu et al., 2021), bisphenol A, a well-known endocrine disruptor (Hayashi et al., 2015), tris (1-chloro-2-propyl) phosphate, a flame retardant (Xia et al., 2021) or urban waters (McIntyre et al., 2014; Young et al., 2018).

## Screening for hair cell protectors

Testing for the ototoxicity of a new molecule is quite straightforward, either in zebrafish larvae or in mammalian inner ear. Searching for substances that protect hair cells from cisplatin or aminoglycoside-induced damage is a more complex matter. These molecules will have to fulfill a series of requirements: must have a protective effect without compromising anti-cancer or anti-microbial activities, be devoid of other toxic effects on their own, and be able to reach hair cells in the mammalian inner ear after systemic administration, eliminating the need for intratympanic administration.

Knowing that damage to hair cells is exerted through dysfunctional mitochondria and ROS production, a number of drugs among the antioxidant class and ROS scavengers have been proposed for the treatment or prevention of ototoxicity: D-methionine, N-acetylcysteine, selenium compound (Ebselen), alpha lipoic acid, coenzyme Q10; however, none of them have performed well in clinical trials (Hammill and Campbell, 2018). Therefore, the need for effective otoprotectants exists, and the hair cells of the zebrafish larvae are a useful model to speed up that goal (Ton and Parng, 2005). Some of the mentioned antioxidant compounds have been positively tested against ototoxins in zebrafish larvae, like glutathione, allopurinol, N-acetylcysteine, D-methionine (Ton and Parng, 2005), L-Serine (Monroe et al., 2021), a curcuminoid (Monroe et al., 2018), flavonoids, like naringin (Li et al., 2021) or quercetin (Lee et al., 2015; Hirose et al., 2016), a small peptide (Xiao et al., 2018), or ferrostatin (Hu et al., 2020).

The small size of zebrafish larvae, and the large number of them that can be obtained, have made possible unbiased systematic approaches to search for otoprotective agents among large collections of chemical compounds. Such studies are not practical using other model systems. Owens et al. (2008) screened 10960 compounds from the Diverset E library against neomycin toxicity. One of the two positive hits, initially called PROTO-1, was further chemically refined following a structure-activity relationship program and, after more than 300 modifications, ORC-13661 was obtained; this compound was orally administered and successful in protecting hair cells in rats treated with amikacin (Chowdhury et al., 2018). ORC-13661 has been shown to be a mechanotransduction channel blocker, protecting hair cells against both aminoglycosides and cisplatin-induced damage, unlike the original compound (Kitcher et al., 2019).

As the process to introduce a new drug into clinical practice can be very long, it is worthy to screen approved drugs for a novel use. Ou et al. (2007) screened a library of 1042 FDA-approved compounds against neomycin toxicity. Seven compounds were found to be effective in zebrafish hair cells, and two of them were tested on neomycin-treated mouse utricle preparations; one was toxic and the other one, tacrine, an anticholinergic agent, showed protective effects. Re-evaluation of the molecules that showed lesser degree of protection in this screen, revealed that eight of them shared a common quinoline scaffold (Ou et al., 2012). Quinoxaline, another quinoline derivative, was found to provide protection against cisplatin, but only partially against aminoglycosides (Rocha-Sanchez et al., 2018). An additional set of 68 quinoxaline derivatives was tested, finding one of them effective against both cisplatin and aminoglycosides in zebrafish larvae and neonatal mouse cochlear explants (Zallocchi et al., 2021). This molecule prevented the ototoxic effects by inhibiting NF-κB, upstream of ROS production (Zallocchi et al., 2021).

We have mentioned earlier that there are differences in the toxic effects and death pathways induced of different aminoglycoside antibiotics (Coffin et al., 2013b). Thus, a molecule found to protect from one aminoglycoside may not be effective against another one. Vlasits et al. approached this problem by screening a library of 640 FDA-approved natural compounds against three aminoglycosides: neomycin, gentamicin and kanamycin, and also cisplatin (Vlasits et al., 2012). Ten of them protected from at least two of the ototoxins, but only two of those were effective against aminoglycosides and cisplatin: bezamil, a diuretic, and paroxetine, a selective serotonin reuptake inhibitor. Recent not yet per-reviewed data point to a role of environmental serotonin in hair cell function (Desban et al., 2022), and several serotonin receptors are expressed in the mammalian inner ear (gEAR Expression Analysis Resource, Hertzano and Mahurkar, 2022).

A similar approach, using neomycin and gentamicin, was used to screen a library of 502 natural products (Kruger et al., 2016). They found four otoprotective molecules that shared a common structure, being derivatives of bisbenzylisoquinoline (Kruger et al., 2016), and nine further derivatives of one of them, berbamine, offered protection against both types of aminoglycoside (Hudson et al., 2020).

Using a more comprehensive strategy using zebrafish larvae and mouse cochlear cultures, Kenyon et al. screened the 10240 compounds from the Life Chemicals Diversity Set. First against neomycin and then against gentamicin in zebrafish larvae, obtaining 64 otoprotective molecules, of which 20 were effective protecting mouse cochlear cultures and only eight of them did not damage hair bundles (Kenyon et al., 2021). What these eight molecules had in common was the ability to block the MET channel, the proposed entry pathway into the hair cell for aminoglycosides (Hailey et al., 2017). In fact, the same group had performed a previous screen focused on a set of 160 ion channel modulators and found 13 positive hits active in zebrafish larvae and mouse cochlear cultures, six of which are MET channel blockers (Kenyon et al., 2017). Therefore, temporary blocking the MET channel to prevent the entry of aminoglycosides on hair cells seems a promising strategy to prevent their ototoxic effects during the treatment course, in order to limit the expected loss of hearing and balance.

Fewer studies have focused on identifying molecules that can protect from cisplatin-induced hair cell damage in zebrafish larvae. The screening of the 1280 compounds from the Sigma LOPAC library against cisplatin toxic effects on both hair cells and pronephros found 22 molecules effective on both assays, and two of the more active ones were members of the dopaminergic pathway, dopamine and L-mimosine, an inhibitor of dopamine β-hydroxylase structurally related to dopamine (Wertman et al., 2020). Interestingly, the known antioxidant N-acetylcisteine was found to be otoprotective, but not nephroprotective (Wertman et al., 2020).

The rise of zebrafish as an animal model has been driven by the ability to perform genetic screens looking for genes affecting embryonic development (Driever et al., 1996; Haffter et al., 1996), including the lateral line (Whitfield et al., 1996). This approach was also taken in search for genes than could prevent aminoglycoside-induced hair cell damage (Owens et al., 2008; Stawicki et al., 2016). The first of these screens yielded five recessive mutations that conferred resistance to neomycin-induced damage and two of the genes were identified: cc2d2a, a cilia protein (Owens et al., 2008), and gcm2, a transcription factor important for the development of ionocytes in the skin of teleosts, mentioned previously (Stawicki et al., 2014). The second screen resulted in the identification of six additional cilia proteins, revealing different roles for intraflagellar transport proteins and transition zone proteins in aminoglycoside-induced hair cell damage (Stawicki et al., 2016).

## Limitations on the use of zebrafish for hair cell toxicology

Studies conducted over the past 20 years have shown that zebrafish larvae are a useful model for hair cell toxicology, due to the easy access to their hair cells to study *in vivo* ototoxin mechanisms, and their small overall size, making possible high throughput screens. However, we have to consider the limitations of the model as well. First, lateral line hair cells are homologous to mammalian inner ear hair cells, but there are no known differences among them as in mammals, where we can distinguish inner and outer hair cells in the cochlea, or type I and type II hair cells in the vestibular system. These different hair cells have been shown to present distinct sensitivities to ototoxins, even between regions of the same epithelia (Forge and Schacht, 2000; Hirvonen et al., 2005; Fettiplace and Nam, 2019). Second, lateral line hair cells are exposed to the environment and drugs can readily reach them. Mammalian inner ear hair cells are isolated from the rest of the body by the blood-labyrinth barrier (Glueckert et al., 2018). Current therapies to prevent hair cell loss are based on invasive methods to introduce the drug into the inner ear (Patel et al., 2019). Related to this fact, otoprotective drug doses effective on zebrafish lateral line hair cells could be different to the levels of that drug found in plasma when used in mammals. Third, lateral line hair cells have the ability to regenerate rapidly after the initial insult, preventing the study of ototoxin exposures beyond 24 h, while ototoxicity in patients takes longer periods to develop.

Despite these limitations, research on ototoxicity using zebrafish hair cells may offer new insights into the underlying mechanisms that could be translated into a way to prevent it. On the other hand, it is hoped that at least one of the otoprotective molecules that have been discovered using zebrafish lateral line hair cells reaches the stage when it could be used in clinical settings to preserve the hearing and balance of patients, making an impact in their lives. The recent elucidation of the structure of the main component of the mechanotransduction channel will surely bring a new wave of focused screens (Jeong et al., 2022), as well as the implementation of machine and deep learning to discover new ototoxins (Zhou et al., 2014; Huang et al., 2021) or new otoprotective agents to be tested rapidly on zebrafish larvae.
